# Integrating ER and Mitochondrial Proteostasis in the Healthy and Diseased Heart

**DOI:** 10.3389/fcvm.2019.00193

**Published:** 2020-01-15

**Authors:** Adrian Arrieta, Erik A. Blackwood, Winston T. Stauffer, Christopher C. Glembotski

**Affiliations:** Department of Biology, San Diego State University Heart Institute, San Diego State University, San Diego, CA, United States

**Keywords:** mitochondria, proteostasis, UPR, endoplasmic reticulum, protein folding

## Abstract

The integrity of the proteome in cardiac myocytes is critical for robust heart function. Proteome integrity in all cells is managed by protein homeostasis or proteostasis, which encompasses processes that maintain the balance of protein synthesis, folding, and degradation in ways that allow cells to adapt to conditions that present a potential challenge to viability (1). While there are processes in various cellular locations in cardiac myocytes that contribute to proteostasis, those in the cytosol, mitochondria and endoplasmic reticulum (ER) have dominant roles in maintaining cardiac contractile function. Cytosolic proteostasis has been reviewed elsewhere (2, 3); accordingly, this review focuses on proteostasis in the ER and mitochondria, and how they might influence each other and, thus, impact heart function in the settings of cardiac physiology and disease.

## ER Proteostasis

**Figure 1:**
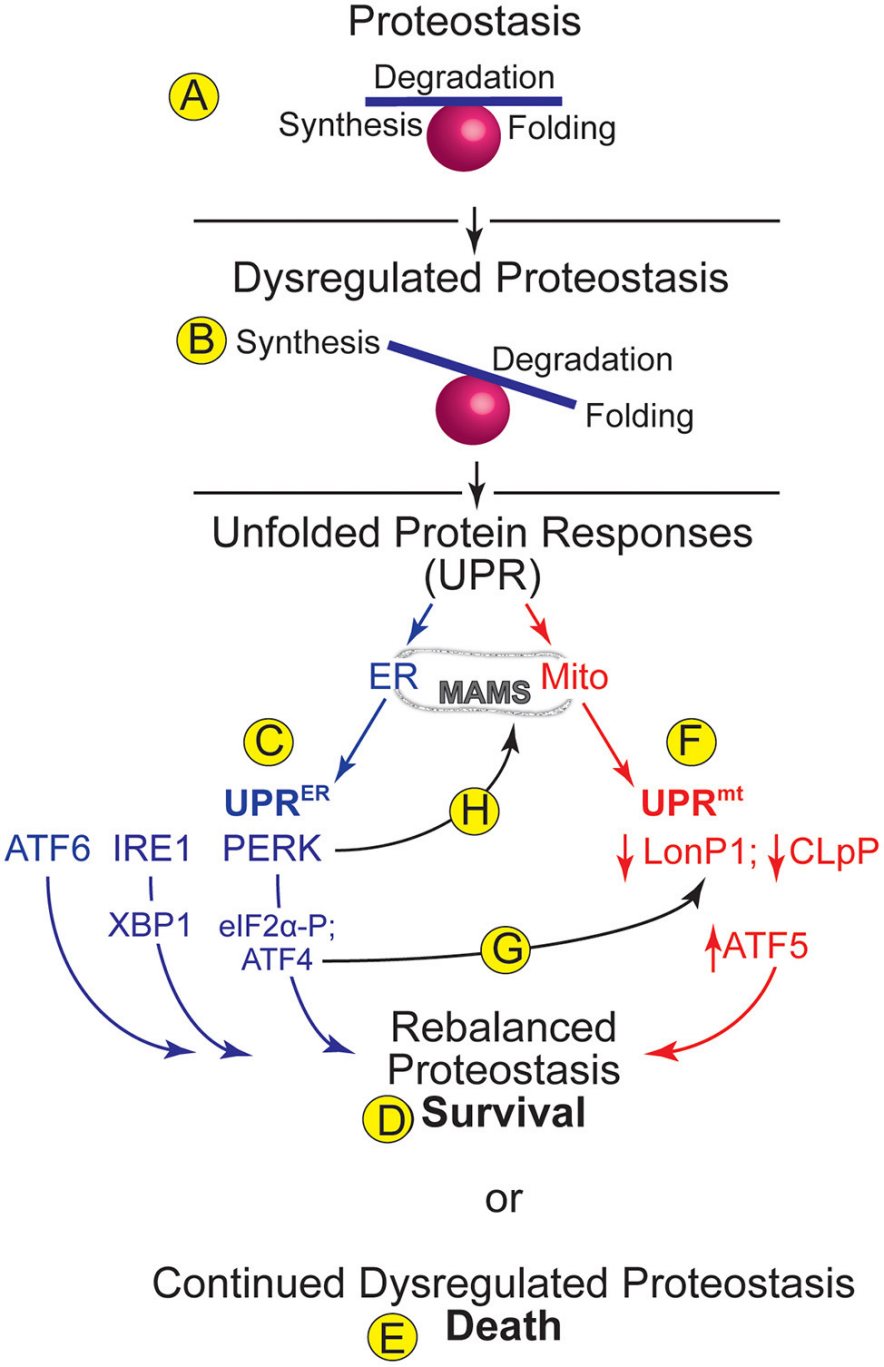
ER and Mitochondrial Proteostasis- **(A)** Proteostasis encompasses processes such as protein synthesis, degradation, and folding. A balance amongst such processes supports optimal proteome integrity. **(B)** Dysregulated proteostasis occurs when environmental conditions, including cardiac pathology, cause an imbalance in these processes, which activates adaptive compensatory responses, such as the unfolded protein responses (UPRs) in various organelles. **(C)** The UPR in the endoplasmic reticulum (ER) is called the UPR^ER^. Increased levels of misfolded proteins in the ER activate three ER transmembrane proteins, ATF6, IRE1, and PERK, which cause increases in the transcription factors ATF6, XBP1, and ATF4, which together regulate genes designed to rebalance ER proteostasis. PERK also phosphorylates eIF2a, which arrests translation of most mRNAs, thus relieving the protein-folding burden on the ER and allowing for cell survival **(D)**. **(E)** Continued dysregulation of proteostasis leads to chronic activation of the UPR^ER^ proximal sensors and cell death. **(F)** The UPR in mitochondria (mt) is called the UPR^mt^. The levels of the mitochondrial proteases, LonP1 and CLpP decrease upon dysregulation of mitochondrial proteostasis. Decreased LonP1 and CLpP contribute to increasing the level of the transcription factor, ATF5, which regulates genes designed to rebalance mitochondrial proteostasis. **(G)** A potential integration point between the UPR^ER^ and the UPR^mt^ is the ability of the UPR^ER^-activated transcription factor, ATF4 to increase expression of the UPR^mt^ protease, LonP1. **(H)** Another potential integration point between the UPR^ER^ and the UPR^mt^ is the ability of PERK to tether the ER to mitochondria at contact sites called mitochondrial associated membranes (MAMS).

Most secreted and membrane proteins are made in the ER, making it a major site for proteostasis (4, 5). Moreover, the specialized ER in cardiac myocytes, which includes the sarco/endoplasmic reticulum, is responsible for contractile calcium handling (6–9), and most of the proteins that are required for this important function of the heart are made at the ER (10, 11). Thus, ER proteostasis in the heart, and in particular in cardiac myocytes, is critical for proper cardiac function. ER proteostasis requires an environment that optimizes a balanced synthesis, folding and degradation of proteins made in this location (Figure 1A). Conditions, including cardiac pathologies can perturb the ER environment in ways that decrease the efficiency of ER protein folding, leading to the accumulation of potentially toxic misfolded proteins, which imbalance and dysregulate proteostasis, leading to activation of the unfolded protein response (UPR) (Figure 1B) (12). Misfolded proteins in the ER are detected by 3 well studied transmembrane proteins, ATF6 (activating transcription factor 6), IRE1 (inositol requiring enzyme 1) and PERK (protein kinase R [PKR]-like ER kinase), each of which exhibits a unique mechanism of activation in response to the accumulation of misfolded proteins in the ER; thus, ATF6, IRE1, and PERK initiate three different but complementary branches of the ER unfolded protein response (UPR^ER^) (Figure 1C) (13). The UPR^ER^ can also be activated by other cellular stresses that could impact proteostasis, or may be independent of it, including changes in ER lipid content (14), hypoxia (15, 16), growth stimuli and reactive oxygen species (17). Thus, while ATF6, IRE1 and PERK were originally found to all be activated by overt ER protein misfolding, it is now clear that they are activated differentially by different pathophysiological stresses and, as a result, the downstream signaling events initiated by each stress are different yet complementary, as far as their ultimate effects on cell function.

In terms of the canonical role for ER stress, initially, UPR^ER^ signaling is designed to restore proper protein folding to the ER, constituting an adaptive return to proteostasis and cell survival (Figure 1D). This restoration takes place at many levels, including enhanced expression of chaperones to facilitate protein-folding, increases in the rate at which misfolded proteins in the ER are degraded through a process called ER associated degradation (ERAD) (18), and decreases in translation of mRNAs that encode proteins that are not required for the restoration of ER proteostasis (19, 20). However, if these complex initial events of the UPR^ER^ are not sufficient to restore proteostasis, then continued dysregulation of proteostasis leads to chronic activation of the proximal sensors and cell death (Figure 1E), and is thus considered maladaptive (21).

## ER Proteostasis in Cardiac Pathology

**Figure 2:**
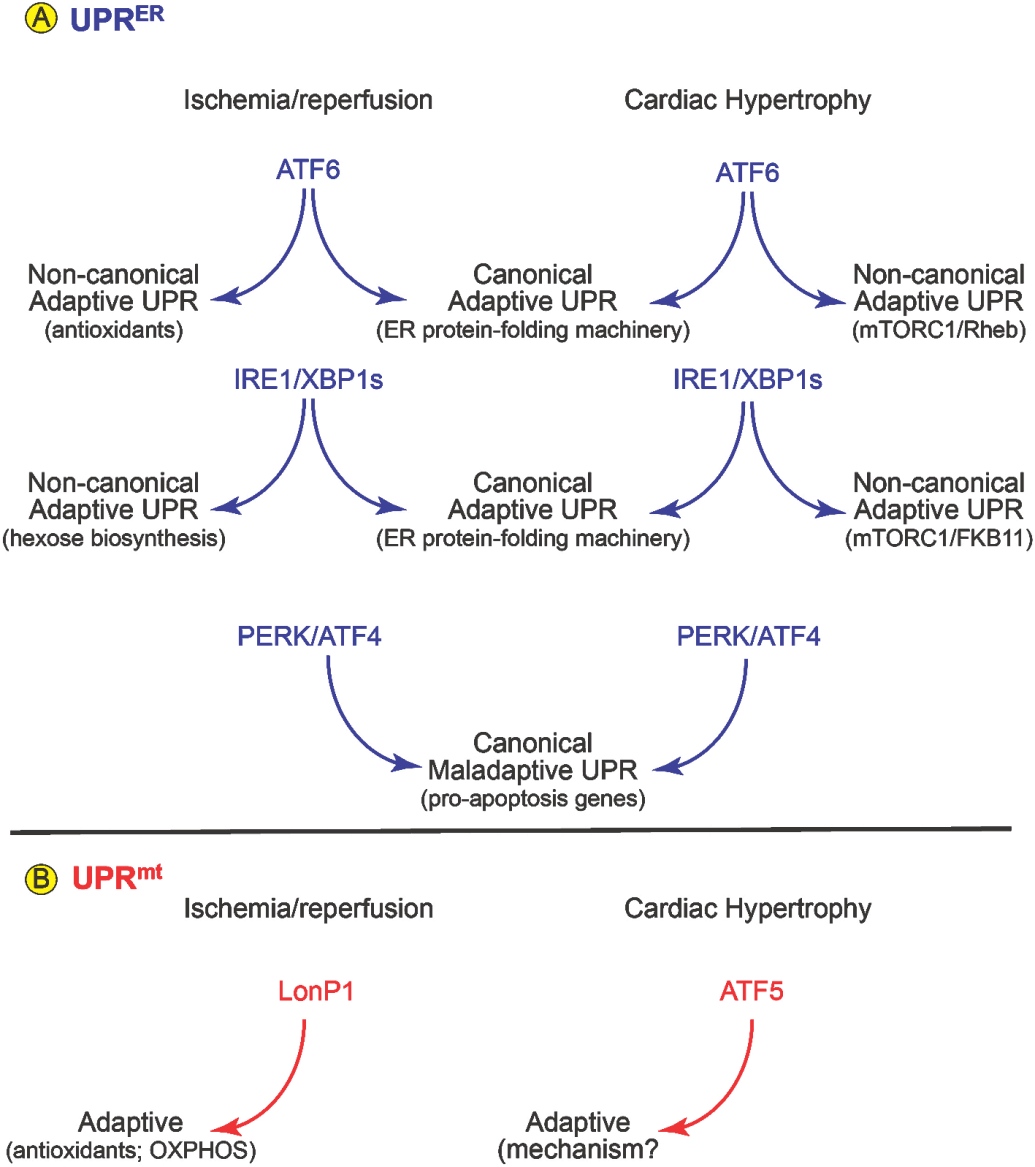
Roles for the UPR^ER^ and UPR^mt^ in Cardiac Pathology- **(A)** In mouse models of cardiac ischemia/reperfusion and pathological cardiac hypertrophy there is evidence for activation of all three arms of the UPR^ER^. (Center) Upon activation each arm of the UPR induces canonical ER stress response genes which support protection for ATF6 and IRE1/XBP1s and damage for PERK/ATF5. However, the ATF6 and IRE1/XBP1s arms of the UPR^ER^ also induce non-canonical gene programs that foster protection in the heart (left and right). **(B)** In mouse models of cardiac pathology the LonP1 and ATF5 aspects of the UPR^mt^ are activated and both are protective in these disease settings.

A number of studies have demonstrated important roles for the UPR^ER^ in the heart; most of these studies have focused on examining ER proteostasis in cardiac myocytes. For example, the ATF6 branch of the UPR^ER^ is mainly adaptive and can protect the heart during pathophysiological maneuvers involving ischemia/reperfusion (I/R) and pressure overload in mice (13, 17, 22–26), the latter of which mimics hypertension and stimulates pathological growth of the heart. The adaptive effects of ATF6 are considered to be largely due to its abilities to serve as a transcription factor following its activation (17, 25, 27). Consistent with this are findings that the genes induced by ATF6 as part of the UPR^ER^ are known to participate in adaptive restoration of proteostasis in the heart by inducing canonical adaptive UPR genes, such as those proteins that constitute the ER protein-folding machinery (Figure 2A), thus serving protective roles (28). Surprisingly, upon activation, ATF6 has been shown to induce a number of genes not previously thought to be involved in restoring ER protein folding capacity. For example, the induction of catalase during cardiac I/R (29), was a surprise, since catalase is not an ER protein, nor is it known to be involved in restoration of ER proteostasis. However, in that study it was shown that ATF6 can transcriptionally induce catalase during I/R and, as a result, catalase neutralizes damaging reactive oxygen species that accumulate during reperfusion, which decreases myocardial damage, thus describing catalase as a non-canonical adaptive UPR gene (Figure 2A, ischemia/reperfusion). In another study, it was shown that during acute pressure overload, ATF6 is necessary for the initial growth of the heart, which is an adaptive effect (17). In that study, using mice in which ATF6 was deleted specifically in cardiac myocytes, it was shown that ATF6 transcriptionally induces the small GTP binding protein, Rheb, which is an activator of mTORC1 (Figure 2A, Cardiac Hypertrophy), a well-studied pathway responsible for myocardial growth during development and pathology, thus describing Rheb as a non-canonical adaptive UPR gene induced during cardiac hypertrophy. Another study involved global deletion of ATF6 and showed that after acute pressure overload compensatory hypertrophy was impaired by ATF6 deletion, while ATF6 deletion led to increased hypertrophy and impaired function after chronic pressure overload (25). It is interesting to note that while it was not studied in the context of activating ATF6, Rheb-mediated mTORC1 activation has been shown to suppress mitophagy, which is generally considered adaptive during cardiac pathology (30–32), suggesting that mTORC1 activation via Rheb is not always adaptive in the heart.

Other branches of the UPR^ER^ have also been implicated in the adaptive responses of the heart of pathological stress. For example, in mouse hearts ischemia/reperfusion activates the IRE1 branch of the UPR^ER^, leading to the formation of an active transcription factor, XBP1, which protects cardiac myocytes from I/R damage, in part, by inducing canonical adaptive UPR genes (Figure 2, IRE1/XBP1s; canonical) (33). In that study it was subsequently shown that XBP1 protects the mouse heart from I/R damage in a non-canonical manner by transcriptionally inducing key genes responsible for the hexosamine biosynthetic pathway, which is required for protein O-GlcNAcylation (Figure 2, I/R non-canonical). Cardiac I/R was shown to increase protein O-GlcNAcylation in the hearts of mice (34), suggesting that O-GlcNAcylation is protective. Moreover, inhibition of O-GlyNAcases increased mitochondrial OXPHOS enzyme activities, implying that this is one way that O-GlcNAcylation might be protective (35); however, the mechanism by which XBP1-mediated protein O-GlcNAcylation results in cardioprotection remains unclear. In terms of heart failure, it was shown that XBP1s stimulates adaptive cardiac growth through activation of mTORC1, which is mediated via FKBP11 (FK506-binding protein 11), a novel transcriptional target of XBP1s, thus describing a non-canonical protective for IRE1/XBP1s in pathological hypertrophy (Figure 2A, IRE1/XBPs cardiac hypertrophy) (36). It has also been shown that in a mouse model of heart failure with preserved ejection fraction (HFpEF), activation of IRE1 is deficient and restoration of activated XBP1 ameliorated the HFpEF phenotype (37). While this study indicates that IRE1 and perhaps the transcription factor, XBP1, protect against the development of HFpEF, the genes that are responsible for this protection have not been identified.

The PERK branch of the UPR^ER^ has also been studied in the heart. In many tissues, including the heart, PERK is known to be involved in numerous signaling pathways, one of which leads to activation of the transcription factor, ATF4, which increases expression of the pro-apoptotic transcription factor, C/EBP homologous protein (CHOP) (38). Since apoptosis is a major contributor to the decline in cardiac function observed during heart failure and other cardiac pathologies (39), and since CHOP expression is increased in experimental models of heart disease (40), several studies have focused on the effects of CHOP gene deletion in the mouse heart. For the most part, those studies have shown that PERK-mediated induction of CHOP in the ischemic or hypertrophic heart exacerbates cardiac pathology, in large part by increasing cardiac myocyte dropout by apoptosis (41). However, other studies that examined the effects of PERK deletion suggest that PERK is adaptive in the setting of pressure overload induced heart failure (42) (Figure 2A, PERK/ATF4). Studies such as these demonstrate the complex nature of the UPR^ER^, indicating that depending on the circumstances, the UPR^ER^ can be adaptive or maladaptive.

## Mitochondrial Proteostasis

Many cardiac physiology and pathology studies have focused on mitochondria, as they play an undeniably central role in energy generation in the metabolically demanding cardiac myocyte. Thus, processes that comprise mitochondrial quality control, which encompass proteostasis, biogenesis, dynamics (fusion and fission) and mitophagy, are critical for maintaining cardiac myocyte viability and heart contractile function (43). Among the features of mitochondrial quality control, relatively little is known about mitochondrial proteostasis in the heart. In non-cardiac cell and tissue types, stresses similar to those occurring during cardiac pathology cause the misfolding of mitochondrial proteins, as well as impaired mitochondrial protein import and decreased translation of mRNAs in mitochondria (44). Mitochondrial ATP production is at risk when mitochondrial proteostasis is dysregulated because it often leads to alterations in the relative quantities of the hundreds of proteins necessary for oxidative phosphorylation (OXPHOS) (45, 46). Moreover, an imbalance between nuclear-encoded and mitochondrial-encoded OXPHOS proteins affects mitochondrial proteostasis in ways that extend lifespan in mice and worms (47). In fact, since the mitochondrial proteome comprises proteins made in the cytosol as well as in mitochondria, the proteostasis balancing act that must be maintained in mitochondria is particularly challenging (46). One important first line of defense against mild mitochondrial damage is carried out by several mitochondrial proteases, which contribute to the mitochondrial unfolded protein response (UPR^mt^) (Figure 1F). In the mitochondrial matrix, protein turnover is controlled by three AAA proteases: the soluble mitochondrial Lon protease homolog (LonP1) and mitochondrial ATP-dependent CLp protease (CLpP), and the mitochondrial inner membrane-bound m-AAA protease. In the intermembrane space, mitochondrial protein quality is ensured by the membrane-bound ATP-dependent zinc metalloproteinase, YME1L1, the soluble mitochondrial serine protease, HTRA2, the mitochondrial metalloendopeptidase, OMA1, and the mitochondrial presenilins-associated rhomboid-like protein (PARL). These proteases play a variety of roles, such as degradation of misfolded proteins and balancing various mitochondrial constituents, such as OXPHOS proteins. However, most evidence suggests that Lon1 and CLpP are central to the UPR^mt^, while the other proteases may play roles in other aspects of mitochondrial proteostasis and dynamics (48). Moreover, because of the dire functional consequences of reductions in the quality of the mitochondrial proteome, dysregulation of mitochondrial proteostasis is communicated to various parts of the cell through at least five different pathways, including peptide-derived signaling, mitochondrial backup-signaling, mitochondrial translation control (MTC) loss-induced signaling and the mitochondrial unfolded protein response, UPR^mt^.

Although the UPR^mt^ is beginning to be understood more clearly in mammals (49), much of our knowledge of this process comes from studies of the nematode, *Caenorhabditis elegans*. In fact, UPR^mt^ activation protects *C. elegans* against ischemic injury, further supporting potential roles for the UPR^mt^, in the ischemic mammalian heart (50). A key regulator of the UPR^mt^ is the transcription factor, ATFS-1 in *C. elegans*, which in mammals is ATF5, a transcription factor that is imported into mitochondria in an ATP-dependent manner when mitochondrial function and protein folding is optimal (46, 51). Under such conditions, LonP1 and CLpP proteases degrade ATF5 (52). However, when dysregulated OXPHOS and other stresses lead to dysregulated mitochondrial proteostasis, LonP1 and CLpP are diverted toward degrading those misfolded proteins to minimize their toxic effects; this diversion leads to the accumulation of intact ATF5 (Figure 1F) (46, 49, 52). Upon accumulation ATF5 is then exported from mitochondria to the nucleus where it acts as a transcription factor that induces genes encoding proteins designed to improve mitochondrial protein folding and rebalance mitochondrial proteostasis (Figure 1F), such as HSPA9, LonP1, and YME1L. ATF5 also serves as a communicator of metabolic stress by temporarily limiting the transcription of OXPHOS genes encoded in nuclear and mitochondrial genomes, while simultaneously increasing nuclear encoded gene transcription of all glycolysis components, and this is thought to maintain cellular ATP levels until mitochondrial dysfunction is resolved (45, 53).

## Mitochondrial Proteostasis in Cardiac Pathology

Little is known about the UPR^mt^ in the heart; however, several recent publications have provided initial evidence that the UPR^mt^ is important for optimal cardiac function and recovery from I/R injury, as well as in the setting of pathological cardiac hypertrophy (Figure 2B). For example, using LonP1 transgenic mice, as well as mice that are haploinsufficient for the LonP1 gene, it was shown that this UPR^mt^ protease mitigates cardiac injury during I/R by preventing oxidative damage, in part by rebalancing OXPHOS complex subunit levels in an adaptive manner (54). Moreover, pressure overload in mice was shown to activate the UPR^mt^. Additionally, pharmacologic boosting of the UPR^mt^ reduced cardiac pathology in this model (55, 56). In the same study it was also shown that hearts from patients with aortic stenosis, which is often associated with left ventricular overload, exhibited increased expression of genes associated with the UPR^mt^. In another study, mice in which ATF5 was genetically deleted were used to show that the UPR^mt^ protected the heart against I/R in an ATF5-dependent manner (53). Moreover, in the same study RNAseq results demonstrated the induction of numerous genes in an ATF5-dependent manner during pharmacological induction of the UPR^mt^. While these studies implicate roles for the UPR^mt^ in the setting of cardiac pathology, much remains to be determined about the role of this mitochondrial proteostasis pathway in the heart. Underscoring the need for additional studies is a recent report where it was shown that CLpP, which plays a central role in the UPR^mt^ in *C. elegans*, and thought to be important for the UPR^mt^ in mammals was not required for the mammalian UPR^mt^ (52). In fact, in that study it was found that CLpP contributes to mitochondrial cardiomyopathy, such that deletion of CLpP increased *de novo* synthesis of OXPHOS proteins leading to increased ATP and improved cardiac function in mice. On the other hand, a different study, while not in the heart, but done with C2C12 myoblasts, demonstrated that knockdown of CLpP altered mitochondrial morphology and expression of OXPHOS proteins, reduced oxygen consumption, increased reactive oxygen species and impaired myoblast differentiation (57). Interestingly, in this same study it was shown that knocking down CLpP leads to increases in the phosphorylation of EIF2α, which is a hallmark feature of the UPR^ER^.

## Integrating ER and Mitochondrial Function in Cardiac Myocytes

There is some evidence suggesting that there is a potential for integration between the UPR^mt^ and the UPR^ER^. One important piece of this evidence is the physical linkage between mitochondria and the ER at mitochondrial associated membranes, or MAMs (58). Although physical linkages between mitochondria and the ER were reported beginning in the 1960's, the term MAM was christened by Jean Vance, who identified a function for the mitochondrial-ER contact sites in phospholipid transport between these organelles (59). Subsequently, numerous studies of MAMs have identified the proteins that tether the two organelles, including mitofusin2 (60), as well as important physiological roles for their juxta-positioning, which, in the heart, have been centered mostly around the movement of calcium from the ER into mitochondria (61). In this way, MAMs are responsible for coordinating ER calcium flux with a variety of mitochondrial functions, including the ATP generation, as well as apoptosis and mitophagy (62, 63). More recent studies have implicated specific mitochondrial-ER tethering proteins, such as FUNDC1, as having important roles in maintaining normal cardiac contractility in mice (64–66). Studies outside the cardiac context have shown that there are numerous components of the UPR^ER^ that are associated with MAM structure and function, including ER chaperones, the IP_3_ receptor and PERK, which, if deleted decreases calcium movement from the ER to mitochondria (67). Relatedly, PERK deletion in the heart disrupts calcium signaling in cardiac myocytes in mice, *in vivo* (68).

## Integrating ER and Mitochondrial Proteostasis

While studies on MAMs imply that the proteostasis pathways in these organelles must be integrated, to date there have been no studies in the heart that have addressed the molecular details of such integration beyond those involving MAMs. However, studies of molecular integration points between mitochondrial and ER proteostasis pathways have been done in other cell and tissue types, and the results of such studies could begin to inform us about whether such integration might also occur in the heart and, if so, what the functional consequences of this integration might be in terms of cardiac physiology and pathology. One potential molecular integration point between the UPR^mt^ and UPR^ER^ that has been studied extensively in non-cardiac cells and tissues is the integrated stress response (IRS). The IRS is an elaborate signaling pathway in eukaryotic cells that is activated in response to an array of stresses including hypoxia, amino acid starvation, glucose deprivation, ER stress and viral infection (42, 69, 70). All of these pathways converge on the activation of kinases, such as PERK, which phosphorylate eIF2α on serine 51. In addition to causing global translational repression, a feature that reduces the protein-folding burden on nearly all of cellular proteostasis, eIF2α phosphorylation leads to the preferential translation of some transcription factors that have upstream ORFs in their 5′ UTRs, such as ATF4 (71, 72) and to subsequent changes in gene expression that are adaptive upon acute ATF4 activation, but can culminate in apoptosis and necrosis upon chronic ATF4 activation. Importantly, the PERK/ATF4 signaling axis, which plays a central role in the IRS and UPR^ER^ (38), is also involved in the UPR^mt^ (69). In fact, like ATF4, the ATF5 transcript also has an ORFs in its 5′ UTR, so ATF5 levels also increase upon PERK upon activation of either the UPR^ER^ or the UPR^mt^ (73). PERK-mediated increases in ATF4 enhance the expression of the UPR^mt^ component, LonP1 (Figure 1G) (74). Related to this finding, but somewhat perplexing is the observation that chemical inhibition of LonP1 protease activity using CDDO activates the UPR^mt^, as well as increasing ATF4 mediated gene induction (75), indicating a possible bidirectional regulatory linkage between ATF4 and LonP1. Another finding that could serve as a molecular integration point between the UPR^ER^ and UPR^mt^ was shown in chondrocytes, where the ATF6 family member, BBF2H7, induces typical UPR^ER^ genes, as well as the regulator of the UPR^mt^, ATF5 (76). In an examination of the effects of drugs that dysregulate mitochondrial proteostasis in HeLa, 293T, and COS7 cells, as well as maneuvers that cause mitochondrial proteostatic stress, *in vivo*, it was shown that via activation of the ISR, ATF4 but not ATF5 responds to dysregulated mitochondrial proteostasis and activates the expression of cytoprotective genes (77). Moreover, the PERK/ATF4 signaling axis can affect mitochondrial morphology and functional integrity, presumably having these effects at least partly through regulating mitochondrial proteostasis (78). Thus, it seems possible that through PERK-mediated increases in ATF4 and ATF5, and perhaps through PERKs role as a tether which holds MAMs together (Figure 1H), a function that does not require PERK enzyme activity (79), the UPR^ER^ and UPR^mt^ could be integrated and, in some cases co-activated, which could improve both ER and mitochondrial function during stresses that dysregulate proteostasis in these two organelles.

## Conclusion

The processes that govern mitochondrial and ER proteostasis are of critical importance for the adaptation of eukaryotic cells to environmental changes that risk proteome integrity. Even though the processes involved in mitochondrial proteostasis have gone relatively unstudied in the heart, it seems likely that in combination with those that regulate ER proteostasis, they are critical for cardiac function and, in particular, cardiac myocyte viability and contractility. In light of this, it is apparent that mitochondrial and ER proteostasis, which are regulated by many processes in addition to the UPRs in these organelles, provide fertile opportunities for future studies that could lead to the design of novel therapeutics for treating cardiac pathologies, ranging from ischemic to hypertrophic and dilated cardiomyopathies. Our hope is that this review has brought such potential intervention points to light amongst the heart research community and that it will spawn investigation into these aspects of proteostasis, with an objective of developing much needed new therapies for treating cardiac pathologies.

## Author Contributions

All authors listed have made a substantial, direct and intellectual contribution to the work, and approved it for publication.

### Conflict of Interest

The authors declare that the research was conducted in the absence of any commercial or financial relationships that could be construed as a potential conflict of interest.
